# Medical Gossip

**Published:** 1862-04

**Authors:** 


### Art. IX.—MEDICAL GOSSIP.

Whatever regret we may feel at the removal of St. Thomas's Hospital from the site which it has occupied upwards of six centuries, our regret is amply counterbalanced by the consideration that the great objects of this noble charity may be much promoted by the removal. Such regrets as attach to the more ancient associations of the present hospital may well be solaced by a better site; such as attach to the more modern, by a better building. With an annual revenue of upwards of 30,000*l.*; with a prospective revenue of probably double that amount within a comparatively short period; and with funds in hand, from the purchase of the present hospital and its site, largely exceeding 200,000*l.*, we have a just right to expect that the future St. Thomas's shall not only be the first in construction and arrangement of all metropolitan and provincial hospitals, but also that it shall be at least second to none in the world.

Untrammelled by pecuniary restrictions, guided by the lavish experience of late years in hospital construction at home and abroad, and having among their own medical staff advisers second to none living in the peculiar, yet enviable position in which the Governors are compulsorily placed, we can hardly permit ourselves to doubt that the latter will worthily use the grand opportunity now at their command, but (unhappy word!) certain doubts have begun to affect uneasily the public mind.

It would seem that the question has been mooted among the Governors whether the objects of the charity would not be better carried out if the hospital were moved away from town altogether, although into a locality in the vicinity such as would command a ready access to town by rail. How such a question ever came to arise in connexion with so vast and so essentially a local charity, it is somewhat difficult to understand. Common report says, that certain very plausible sanitary considerations were at the bottom of the matter; but "common sense tells us," as the *Times* very happily said, "that hospitals, the very object of which is to relieve urgent cases, should be easily accessible by day and night." Far be it from us to underrate the sanitary advantages of a suburban site, but it appears to us that the problem is not to secure the best site *per se* for the new hospital, but to secure the best site consistent with the necessities of the people for whom the charity was especially created. No doubt by a removal into the suburbs, the utility of the hospital would by no means be lost. The hospital accommodation of London does not exhaust the sickness needs of its vast necessitous population, and the force of circumstances would compel many to seek distant aid, who were unable to secure help nearer at hand. But is it not probable, that the new hospital would be made largely available for the sickness of a population which had hitherto made comparatively little use of hospitals—in fact, that the new site would create in no small degree a new hospital population, while the population which had chiefly benefited by the charity hitherto would seek the aid more readily obtained at Guy's or the hospitals North of the river? It is idle to expect that any population will seek that help miles away in the suburbs, which can be obtained more readily and with greater comfort close at hand; hence the removal of St. Thomas's would not only have the immediate effect of throwing an additional burden upon other and already sufficiently burdened hospitals, but would prove an actual and serious deprivation to one of the most necessitous portions of the population of the metropolis—a population, in short, for which the hospital was, we presume, originally instituted. We have not referred to the influence which such a removal as suggested would have upon the medical staff of the hospital, and the important medical school attached to it; but it is reasonable to suppose that the distance contemplated would of necessity deprive the hospital of the aid of any man of eminence who possessed the semblance of a practice, if that aid were sought under the present conditions of hospital service (and it is not to be forgotten that it is upon the eminence of the medical staffs that the fame of hospitals alone depends); while it can hardly be questioned that the Medical School would come to an untimely end, or at least, be greatly injured in its utility. What student would undergo the discomfort and additional expense attendant upon the journey to and from a suburban hospital, when Guy's, St. Bartholomew's, Westminster, Middlesex, and other hospitals, offered equal, and some greater advantages, within easier reach? And yet the efficiency of the hospital, as a charitable institution, is in no small degree connected with its School of Medicine.

Another subject of distrust is the fact that the medical staff of the hospital have thought it incumbent upon themselves to address, and at the same time make public (or at least the document has been laid before the public), a memorial to the Governors. In this memorial the physicians and surgeons to the hospital refer with pride to its history, and to that of the medical school attached to it, and they comment upon the "singular and splendid opportunity for increasing the usefulness of the institution," afforded by the impending change. "We see," they say, "that our hospital may be made the very first hospital, and our school the very first school in England." "On the other hand," they add, "we feel that the occasion has its dangers; and the fact which, above all, impresses us is that the course which must now very speedily be decided upon will be one from which there can be no return. If, unhappily," they continue, "any considerable error were made in the placing or planning of the new hospital, the consequence of that error would remain for many generations beyond remedy." They point out how "the intentions of the founders and benefactors of the charity would be almost irreparably defeated, and the priceless boon of a great school of medical science would at the same time be sacrificed, if the new hospital were to be planted in any locality where physicians and surgeons of high metropolitan standing could not be expected to serve it with assiduous attention, or where masses of the labouring population would not have easy access to it for the relief of all their emergencies of sudden illness and injury." They proffer their assistance if the governors "should deem it expedient" to ask their opinion "either on the sites which are offered for their choice, or on the plans of building and establishment which are proposed for their approval." Finally, they suggest that "everything possible should be done to ensure a free and full discussion of the important issues which will be raised," and that the several issues should be made public at least one month before the General Court of Governors will have to decide on them.

There is something portentous about this document. We scarcely dare permit ourselves to doubt, and yet we must doubt. Can it be possible that any serious question involving the future site or construction of the hospital can have been entertained by the Governors without the medical staff, or at least the seniors of that staff, having been invited to aid the Governors in their deliberations? Is it to be believed that the Governors of one of the most important metropolitan hospitals can, even for a brief space, ignore the great truth which has been taught by past hospital management, and which rests upon the face of the question, that the chief source of badly constructed, badly arranged, and badly located hospitals, has been the sacrifice of the medical, the essential requirements of the hospitals to architectural and amateur dilettantism? Is it possible that the Governors have not learned, or have neglected the lesson taught by the history of military hospitals (to mention the most recent and important illustrations of hospital building in the kingdom) since the Crimean war? We can scarcely believe that such should be the case, yet why this memorial of the medical staff? Truly it would be just food for laughter throughout the civilized world, if the Governors of St. Thomas's should, through perverseness, miscarry in their present responsible task, when among the medical staff of their noble charity (to mention specially but one important item of success possessed by them in addition to the great pecuniary and other advantages at their command) they enumerate an authority on sanitary questions second to none in the kingdom,—the distinguished medical officer of the Privy Council.

One of the first acts of the Federals after civil war had broken out in the United States of America, was the formation of a Commission to inquire into the sanitary condition of the gigantic army which had, with a rapidity little short of marvellous, been enrolled in the Northern States. The stern lessons which we had been taught in the Crimean war were not forgotten by the Federals in the time of need, and they hastened to anticipate, as far as practicable, the mischief from which we had so signally suffered in the winter of 1854-55, and our allies, the French, in the winter of 1855-56. The Commission formed was not, curious to say, a Government one. It possessed no legal authority, and no pecuniary support. It was self-constituted, but its object at once secured the approval and co-operation both of the people and the army, and the Government gave it free permission to "inquire and advise." Under the presidency of the Rev. Dr. Bellowes, a doctor of divinity, not of medicine, the Commission commenced its operations, and the energy and completeness with which it pursued them are not the least remarkable characteristics of this anomalous body. The labours of the Commission must have proved of infinite value both to the troops and the Government, and the records of these labours will furnish the most important history of that remarkable force with which the Federals first took the field. We have not yet received the detailed reports of the Commission, but in the meanwhile one or two facts relating to its doings may be culled from the general press. Within the period of seven months after commencing their self-imposed duties, the Commission had, according to the *Times,* received upwards of 400 reports from their subordinate officers, each containing 360 questions and answers; and they had furnished the Government with forty reports* of their own, and distributed throughout the loyal States 150,000 copies of "publications" printed by them. They sought to learn, and learned, everything about the troops—where they came from, how they came, how long they were coming, and what kind of men they were, as well as how they were lodged, fed, paid, clothed, entertained, and doctored. In the months of September and October last, as many

* A few days ago, after an active and protracted search, with every aid from the courtesy of the United States officials in town, we were compelled to come to the conclusion, that, excepting the copies of these Reports apparently in possession of the *Times,* no other copies existed in town. The United States ambassador might possibly, but by no means certainly, have copies, and our Foreign department; but of the general public the *Times* alone had possessed the secret of securing these important documents.

as 200 regiments, every one of which had been raised within a space of six weeks, had been severally inspected and catechised. It was found that no less than seventy-six and a half per cent. of the battalions were mainly constituted by native Americans, and that in five or six per cent. only was the majority formed of German or Irish. The average age of the volunteers was supposed to be a "little below twenty-five," about half the whole number being under twenty-three. The clothing of the soldiers bore inspection well. In ninety-four per cent. of the regiments the men had two shirts each; in eighty-two per cent. they had good over-coats; in seventy-five per cent. they had good body-coats, and to a similar extent they had one good blanket each. The rations were abundant, satisfactory in quality, and the cooking was not complained of. In six regiments only out of the 200 was intoxication notoriously common, while in 163 it was reported to be "very rare." In fifty-seven per cent. of the regiments the men were in the practice of sending home between half and three-fourths of their pay, and in certain regiments numbering 1000 men, as many as 600 soldiers posted a letter per day for weeks together, "a delightful indication of a fact," the Commissioners say, "which should remove all fear of a permanent military despotism in this country."

During the months of August, September, and October the number of sick amounted to about 77 per 1000, and on the Potomac the average mortality through the summer is computed at 3½ per cent.; or at 5 per cent. if the remoter camps are included in the reckoning. The chief sanitary evils noted are the injudicious choice of sites for encampments; insufficient ventilation of tents at night; and although the daily removal of refuse was pretty well attended to, the troops lacked such habits of cleanliness as might have been expected from men of their stamp. "Slovenliness is our characteristic national vice," observes the Commission. The tents were variously supplied with flooring. Sometimes the men slept on wooden boards, sometimes on indiarubber cloth, sometimes on boughs or straw, sometimes on the bare ground. It was noted that the nature of the flooring exercised a marked and curious influence on the liability to disease. Fever prevailed most in connexion with indiarubber blankets, least with straw or boughs. Rheumatism occurred most frequently on boarded floors, least when straw and boughs were used. A boarded floor also disposed more markedly to throat affections, which the men were least obnoxious to when they slept on the ground. Here, however, they were exposed to malaria, against which branches and straw were a protection. Typhus fever, which was more or less prevalent in the camps, instead of declining, increased in frequency on the approach of winter. The Commissioners, however, hit upon the right explanation of this paradox. The cold drove the men "to burrow or seal themselves in their lodgings," hence all the evils of one of the worst forms of overcrowding. Small-pox had created some uneasiness in the army; but the Commission began to vaccinate on a large scale, and 20,000 citizens were subjected to that operation, who would otherwise most probably have dispensed with it. The Commission were at once able to discover when a volunteer regiment had any of the officers of the old United States Army doing duty with it, from the better state of its internal economy—a hint to the officers of our own volunteer force.

The Commission made a special Report after the battle of Bull's Run, and from it we learn that "there was an evident disposition to regard the exhausted physical condition of the men as a chief cause of defeat." Many of the troops had not breakfasted, and others had been pushed forward at a pace beyond their endurance. The inexperience of the officers, and the carelessness and insufficient training of the men, almost necessary consequences of the brief period of time during which the force had been in arms, appeared to be the chief causes of these results. It was asserted at the time of the action that many of the troops threw away their rations before commencing their march, rather than be at the trouble of carrying them.

The Committee for carrying out the arrangements for a local memorial to the late Lord Herbert have determined to erect a statue at Salisbury, and to build a Convalescent Hospital, to be named the Herbert Hospital, on the coast of Dorset, available for Devonshire, Somersetshire, Hampshire, Wiltshire, and Dorsetshire. For the former object upwards of 1000*l.* have been received; for the latter upwards of 3000*l.*

About the commencement of the year a discussion was started by Dr. Gosselin, at one of the sittings of the Academy of Medicine, on the comparative sanitary condition of the hospitals of Paris and London. The discussion quickly extended beyond the walls of the Academy, and has occupied the attention of our neighbours considerably during the quarter. In originating the question, Dr. Gosselin stated that from what he had been told by various foreign surgeons, and also by French physicians who had visited foreign hospitals, there existed a considerable difference in the results obtained in the English and German establishments of the kind compared with Paris. In foreign hospitals patients who had been operated upon were treated with much more care, and many more sanitary precautions were taken with them than in Paris. The sick-wards of the London hospitals were much better aired, being provided with large open stoves, which gave rise to draughts, and consumed the vitiated air of the rooms; patients who were able to walk took their meals in the dining-rooms apart from the sick-wards, an arrangement which greatly diminished the crowded state of the infirmaries, and contributed towards maintaining the purity of the air; the floors were frequently washed, which prevented the accumulation of dust, while the beds had no curtains, whereby miasmatic emanations were better dispersed; and, lastly, the linen department was the object of particular care. Dr. Davenne differed in opinion from Dr. Gosselin, and maintained that the manner in which the Paris hospitals were conducted was far superior to the London system. If the sanitary condition of the latter was better, it was owing to the fact that their population was smaller, because the paupers relieved by the poor-tax were not admitted into them. He then quoted some statistical documents to show that the mortality in the Paris hospitals had been constantly diminishing since the commencement of the present century. Dr. Malgaigne said he would confine himself to the sole question of mortality among patients who had undergone an operation, and quoted certain statistical details published by himself some years ago, showing that the results were lamentable. Adverting to an old report of Dr. Tenon's who had visited the hospitals both of London and Paris, he remarked that the author proposed to limit the sick-wards to the ground floor and first story only, every sick-ward containing 24 beds at the utmost. In 1814, he added, the slaughter-houses of Paris were transformed into hospitals, and the results of this measure afterwards published were curious. The mortality of the French operated on in the common hospitals was 1 in 5, 8, 9; in the slaughter-houses, 1 in 10, 12, 13. The mortality of the foreign soldiers operated on in the hospitals was 1 in 7, 13; in the slaughter-houses, 1 in 10, 19. This showed that the mortality was lowest in the best-aired situations. As to the London hospitals, there was one fact to be taken into account—viz., that the sick admitted into them were much more seriously ill than those admitted into the Paris hospitals. Out of 100 patients operated on in each city, 56 died in Paris, and only 30 in London. Out of 100 amputations of the thigh, 60 ended mortally in Paris, 21 in London, and 19 in Manchester, where the hospitals have the advantage of the country air. Now what was the reason of this enormous difference? Simply, that while in the Paris wards there are as many as 80 beds, there are only 12 in the London ones. The subsequent progress of the discussion has tended to confirm the opinions originally expressed by Dr. Gosselin and Dr. Malgaigne, and the most recent writer on the subject, M. Lefort, is inclined to attribute some influence to the effect of *treatment*, in bringing about the difference in the mortality observed in our own as compared with the Paris hospitals. "If," he says, "we cannot attribute the difference of mortality solely to the [sanitary and other] circumstances to which I have just referred, we then must search for its cause in a more strict general observance of those thousand precautions which, though separately insignificant, become, when conjoined, matters of great importance. According to all that I have witnessed, *treatment* must have its share in this respect. . . . Dressings reduced to very great simplicity, a nourishing, a highly nourishing diet after operations, the employment of tonics and alcoholic stimulants, and opiates even in very full doses, have appeared to me to have a marked influence in lessening mortality." He then refers to the fact that resections of joints, ovariotomy, and other serious operations, are still, from their great fatality in French hospitals, the opprobria of French surgeons, but are familiar to the English surgeon, and attended with the happiest results in English hospitals.

We have recently learned much from our neighbours on the construction of hospitals, and perhaps they may in return obtain some valuable hints from us on their internal economy.

It is to be hoped that the Secretary of State for Ireland will be more fortunate with the Bill, which he recently brought before the House of Commons, for the Registration of Births and Deaths in that portion of the kingdom, than his predecessors had been with similar Bills. On introducing the Bill, he naturally dwelt chiefly upon the importance of a registration such as proposed, in establishing the identity of individuals and titles to property, and pointed out the serious difficulty occasionally arising in those respects from the want of a systematic registration. On the scientific and social importance, otherwise than in its legal aspects, of a registration of births and deaths, we presume no question will now be entertained, and the right honourable secretary passed over the arguments in favour of his measure arising from these considerations; but in detailing the machinery with which he proposed to carry his Bill into operation, he mentioned one or two facts not without interest to the medical profession. Who were to be the registrars, and in what manner were their districts to be defined? Practically, the question who shall be registrars had, it would appear, been the great stumbling-block to previous legislation on the subject. Six different classes of agents had been proposed by whom registration should be carried into effect: 1, district medical officers; 2, postmasters; 3, ecclesiastics; 4, persons nominated by the Lord Lieutenant; 5, persons appointed by boards of guardians, and 6, the constabulary. To devolve the duty upon the postmasters was characterized as absurd; to devolve it upon persons selected by the Lord Lieutenant, would be to place too much power in the hands of one officer in Dublin; that such a civil matter should be entirely entrusted to ecclesiastics would clearly be distasteful to the public; while again, if the registrars were chosen by boards of guardians, they would have to be nominated by the majority, and the minority would then always be at issue with the person elected. Consequently the Secretary resolved these schemes into two, the only two which appeared to him applicable to the circumstances of Ireland. One of these schemes is registration by medical officers, the other by the constabulary. Hypothetically, it might seem to the looker-on that no doubt could be entertained as to the greater fitness of the medical man for the office; practically, however, it would appear, on the showing of the Secretary, that the office is by no means fitted for him. The great thing, he said, was to have the thing done most efficiently, and yet, at the same time most economically. Now, the advantage of using the constabulary as registrars of births and deaths, in preference to the medical officers of unions, is to be gathered from the following facts amongst others. The number of constabulary districts in Ireland is 1570, giving an average area of 20 square miles to each; the number of dispensary districts, on the other hand, is 716, with an average area of 40 miles! So that the registering constables would have districts less than one-half the size of those of medical officers. Again, the average number of families to be visited by each registering constable would be 719, while the number to be visited by each dispensary officer would be 1577. The average number of births and deaths to be registered by each constable would be 200, and by each medical officer, 438. The average yearly remuneration proposed to be given to each constable was 5*l.*, whereas the amount which each medical officer would receive, calculated at the rate of 6d. as his fee upon each entry, would be 10*l.* 19s. "Now," said the right honourable Secretary, "10*l.* 19s. a-year, or about 7d. per day, is hardly enough to induce a medical man of education and position to inform himself of and register every birth and death occurring in a district covering on an average 40 miles. His daily avocations take him away from home for a great part of his time, and he would be obliged to have a deputy. Why, it is a well-known fact that the Poor Law Commissioners have stated that they think the 1s. fee which a medical officer receives for each case of vaccination is hardly sufficient to induce him to do the duty efficiently. How, then, can we expect him, for 6d., to attend to a matter scarcely within the immediate scope of his profession?" How could we expect him to do so unworthy a thing? But we do expect him, under most delicate compulsion, to contribute, in the interest of the State, a highly essential portion of the death registration without fee or reward. "I may mention," said Mr. Secretary, "that we propose to require, as in Scotland, that the medical attendant of the deceased person shall transmit his certificate within seven days from the death."

